# Epidemiology and Changes in Patient-Related Factors from 1997 to 2009 in Clinical Yeast Isolates Related to Dermatology, Gynaecology, and Paediatrics

**DOI:** 10.1155/2013/703905

**Published:** 2013-12-11

**Authors:** Viktor Czaika, Pietro Nenoff, Andreas Glöckner, Wolfgang Fegeler, Karsten Becker, Arno F. Schmalreck

**Affiliations:** ^1^Klinik für Dermatologie, Venerologie und Allergologie, Campus Benjamin Franklin, Charité-Universitätsmedizin Berlin, Hindenburgdamm 30, 12203 Berlin, Germany; ^2^Laboratorium für medizinische Mikrobiologie, Straße des Friedens 8, 04579 Mölbis, Germany; ^3^BDH-Klinik Greifswald GmbH, Karl-Liebknecht-Ring 26a, 17491 Greifswald, Germany; ^4^Institute of Medical Microbiology, Domagkstraße 10, 48149 Münster, Germany; ^5^MBS—Microbiology, P.O. Box 101247, 80086 Munich, Germany

## Abstract

From 1997 to 2009, 1,862 dermatology, gynaecology, and paediatrics (DGP) associated clinical yeast isolates were analysed for species occurrence, specimen origin and type, (multi-) resistance pattern, and testing period. The top seven of the isolated DGP-associated species remained the same as compared to total medical wards, with *Candida albicans* (45%) as most frequent pathogen. However, the DGP wards and DGP ICUs showed species-specific profiles; that is, the species distribution is clinic-specific similar and however differs in their percentage from ward to ward. By applying the “one fungus one name” principle, respectively, the appropriate current taxonomic species denominations, it has been shown that no trend to emerging species from 1998 to 2008 could be detected. In particular the frequently isolated non-*Candida albicans* species isolated in the DGP departments have already been detected in or before 1997. As yeasts are part of the cutaneous microbiota and play an important role as opportunistic pathogens for superficial infections, proper identification of the isolates according to the new nomenclature deems to be essential for specific and calculated antifungal therapy for yeast-like DGP-related infectious agents.

## 1. Introduction

Superficial fungal infections are often chronic and recurring. It has been estimated that approximately 15% of the population has fungal infections of the skin (tinea pedis or athlete's foot) or nails (onychomycosis) or of the feet. These infections are common in older children and adults [1]. Distal subungual, proximal, subungual, and white superficial onychomycoses are usually caused by dermatophytes, but *Candida* spp. may be present in all types in less than 1% of these cases [2]. In the past, yeasts are thought to be simply skin contaminants [3]; however, yeasts and nondermatophyte moulds may also cause toenail onychomycosis [4–8]. A higher proportion of yeasts is generally found in onychomycosis, where dermatophytes (68%), yeasts (29%), and moulds (3%) are the most causative fungal pathogens [9]. Some *Candida* spp. causing onychomycosis were reported to be partly resistant to oral antifungal agents (AFAs). In patients with chronic mucocutaneous infections, the main yeast pathogen is *Candida* (*C.*) *albicans,* but *C. tropicalis*, *C parapsilosis*, *Issatchenkia *(*I.*)* orientalis*, and *Meyerozyma* (*M*.) *guilliermondii *may also contribute to these infections [10].

It has been suggested by Clayton and Noble [11] that the spread of yeasts in the hospital ward occurs in a similar way to the spread of *Staphylococcus aureus*. In addition, the carriage rates of yeasts on the skin in hospital patients appear to be higher than those in the nonhospital population [11]. As cutaneous sites may act as common sources of infection, the ability of patients to disseminate bacteria and yeasts is to a greater extent due to the colonization of the skin, and to the fact that such patients liberate more particles of the skin than persons with a clinically normal skin [12]. As with *C. albicans,* the non-*C. albicans Candida* (NCAC) infections within 276 NCAC (14%) of 1,972 *Candida* isolates, as reported by Somerville [13], contribute to a significant amount to the hospital acquired infections.

Species distribution within the not-*Candida* yeasts (NCY), for example, of *Trichosporon *spp., which are emerging in Asian countries [14], may be strongly influenced by antifungal agent use [15]. Parallel to the increasing rate of dermatological infections by NCAC and NCY species, an increasing number of these usually opportunistic pathogens [14, 16–19] are isolated of critically ill patients [20, 21], from the oral cavity [22, 23], in pulmonary infections [24], from cutaneous (intertriginous, paronychianous) and mucocutaneous (vulvovaginal, balanitinous) infections [25–27], genitourinary tract infections [28, 29], and in the intensive care unit [30]. The most common fungal infections in infants and children are mucocutaneous candidiasis, pityriasis versicolor, tinea corporis, tinea pedis, and tinea capitis [27]. *Candida* colonization has a considerable prevalence among paediatric and neonatal patients [31–35]. Preterm newborns in the paediatric ICU where besides vaginal delivery, low birth weight, and low gestational, age can be considered as risk factor for colonization [36]. Oropharyngeal candidiasis (thrush) may start as early as seven days after birth, with an incidence in infants of 5% to 10% depending on the population studied [31, 37, 38].

Aside of the changing epidemiology of classical and emerging human fungal infections [39], the incidence of atopic dermatitis (AD), a multifactorial disease in which both hereditary and environmental factors play a role, has been increasing. The worldwide prevalence of AD is about 10%–20% in children and 1%–3% in adults [40–42]. In a total of 241 samples in a Lithuania clinic of patients with clinical diagnosis of AD exacerbation, most isolated genera were 27.4% *Candida*, 6.6% *Malassezia*, and 2.9% *Rhodotorula mucilaginosa*. The species most frequently isolated in child and adult groups were *Debaromyces hansenii*, *C. pelliculosa*, *C. parapsilosis*, and *Malassezia furfur* [42–44].

Therefore, precise strain identification and knowledge of the epidemiology of *Candida* and NAC species are essential and are of great advantage in making and optimizing treatment decisions, especially when the phylogenetic relationship of the expected isolates [45] is additionally considered.

The aim of this reevaluation, after renaming the isolates according to their currently valid taxonomic denomination, was to evaluate the distribution and occurrence rate of the relevant yeast species, isolated from dermatology, gynaecology, and paediatric (DGP) patients. By using the “new nomenclature” this study should build a valid basis for the comparison of recent and future fungal epidemiological surveys. The actual susceptibility profile and its possible changes during the isolation period (1998–2008) of the 1,862 clinical DGP-yeast isolates, tested against frequently used azole antifungal agents in this area, are given in a corresponding paper [46].

## 2. Material and Methods

The 1,862 clinical yeast isolates (Table 1) were derived from dermatology, gynaecology, and paediatric wards from German University hospitals in Berlin (Charité), Dresden, Leipzig, Münster, and Munich (Ludwig-Maximilians-Universität München, and Technische Universität München) starting at the end of 1997 until February 2009, in the framework of 4 multicenter studies (MCS) [49–51]. The few numbers of strains (*n* < 40) of the years 1997 and 2009 were added to those isolates of 1998 and 2008, respectively. Therefore, the time period throughout this paper is referenced as 1998 to 2008. In addition, as no MCS were performed from 2005 to 2007, respectively, no DGP clinics participated; therefore isolates from this time period are missing. For comparison and possible trend recognition additionally, and with respect to the number of isolates, two similar test periods (1998–2001 and 2002–2008) have been set up out of the total study-period.

Identification and differentiation of the isolates were performed using methods routinely employed at the microbiology/mycology laboratories of the participating test centres. Confirmatory identification was made for unusual or not-identified species by FTIR and/or PCR at the appropriate reference laboratory of the individual multicenter study. As the “one fungus one name” principle is effective since 2013 [52, 53], the current valid names for the appropriate species were applied throughout this paper as published in SpeciesFungorum [47], respectively, in MycoBank [48].

Susceptibility testing of these isolates was performed by microdilution against relevant azole antifungal agents, as described in the corresponding paper [46].

To ease the evaluation and setting of tables the patient-related factors such as clinical specialities (different wards), origin and type of specimen were merged and subsumed in large groups, for example, *aspirates* (transtracheal, limbic, materials from punctures, e.g., abscess, bursa, pericardial, pleura, rectum, and pus); *catheters* (indwelling, vascular, venereal, ports, and anaesthetic tube); *sterile fluids* (sterile body fluids, liquor, dialysates, BAL, tracheal secrets, pleura, lachrymal, synovial, and serum, except blood and urine); *solid* (sterile) *materials* (tissue/lung tissue, bone-marrow, throat discharge/sputum, abscess, spleen, bone, liver, stomach, and ear); *devices* (contact lenses, artificial joints, dialysis access, haemodialysis grafts, cardiac devices such as heart valves, pacemakers, ICDs, VADs, central nervous system devices, penile implants, vaginal sponges, diaphragms, and intrauterine devices); *dermatological materials* (skin scrapings, nails/nail scrapings, plucked hair, dandruff, and scales); *gynaecological materials* (scrapings, genital-, prostate secrets, ejaculate), *urine* (mid-stream, punctuate, and catheter); *external* (external clinics, doctors office, and external laboratory); *general medicine* (allergology, angiology, bronchoscopy, dialysis, endocrinology, gastroenterology, geriatrics, internal medicine, emergency room, endocrinology, nephrology, pneumology, policlinics, psychiatry, rheumatology, rehabilitation centre, standard care, HIV centre, and tropical medicine); *swabs* (surfaces, all body parts, wounds, skin, stoma, ear, and bone), *surgery* (abdominal, aesthetic, general, heart, plastic, vascular, and neurosurgery).

## 3. Results and Discussion

### 3.1. Species Distribution

**Figure 1:**
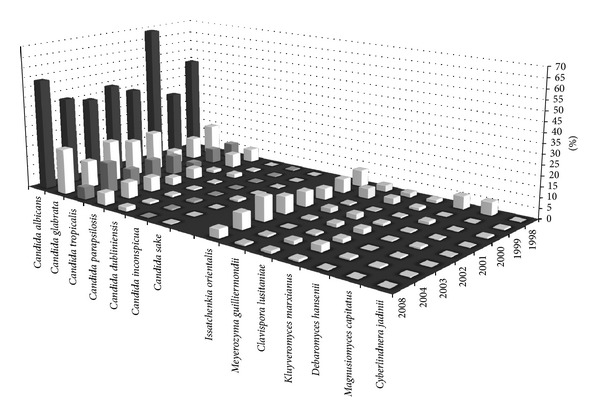
Distribution of the most frequently isolated DGP-related *Candida* and non-*Candida* species from 1998 to 2008.

The distribution according to their isolation frequency of clinical *Candida* and NCY species isolated during the MCS from 1998 to 2008 of DGP patients is shown in Table 1.* Candida* and NCY species most frequently recovered are displayed in Figure 1 by their distribution of the year of isolation. As the “one fungus one name” principle is effective from the beginning of 2013 [51], the current valid taxonomic denominations for genera and species [47, 53] were listed in parallel to the reported species names by the test centres (Table 1). The “new” genus/species names were used throughout this paper. As consequence of the species-renaming, a substantial reduction in species assigned to the genus “*Candida*” and a raise of “new” species occurred. Thus, of the *Candida* clade [37] of 19 *Candida* species reported, nine (47%) had to be renamed, and the name-changes were not only restricted to the *Candida* species. The “new” (“emerging”) taxons, partly called before “not-*Candida albicans Candida*,” “*not-Candida yeasts*,” or “cryptic” pathogens [16–18, 34, 54–58], were already widely present at the beginning of and found throughout the German multicenter studies [49–51].

Despite the “new” taxonomy, *Candida* species remain to be the most frequent pathogens with *C. glabrata*, *C. parapsilosis*, *C. tropicalis*, *C. inconspicua*, and on top *C. albicans, *causing the majority of fungal infections in the DGP area [25, 35]. Although the most prevailing species in all three wards (DGP) is *C. albicans,* its percentage of occurrence differs significantly (Table 2). *Candia krusei* and *C*. *guilliermondii*, which also belong to the class of the 8 most frequent agents to cause fungal infections, are now to be found under the taxonomic designations *Issatchenkia orientalis* and *Meyerozyma guilliermondii*. When compared to the isolation rates of clinical yeast isolates from total medical wards (Table 1; survey 2008), the ranking of isolated DGP-related species differs somewhat; however, the top seven of the isolated species remained the same, as also shown by the corresponding data from a recent dermatology ward survey (Table 1). Interestingly, the species distribution of the 10 years study is similar to the survey conducted in 2008 for the general yeast distribution in Germany (Table 1), whereas the distribution of the dermatophytes from 1998 to 2008 (Table 2) is similar to the survey from 2003 to 2011 within a dermatology unit (Table 1). The predominance of yeasts in both surveys and the study agrees with other studies performed elsewhere [10, 59], with *C. parapsilosis* as the second frequent dermatology associated pathogen. As nationally/internationally reported for invasive fungal diseases (IFD) and *Candida* blood stream infections [54, 55, 58–64], the isolation frequency of the major pathogens from the DGP wards (Tables 1 and 2) followed the change of IFD in Europe [65] and mirrors the changing occurrences of *Candida* and NCY strains. Thus the “valid” *Candida* species were most prevalent among the DGP strains with 76.5% of the total isolates (Tables 1 and 2), including *Candida albicans* (59% thereof; 45% of total isolates), followed by the ascomycetous NCY species (17%), and the basidiomycetous yeasts with 3%. Besides *C. albicans* (D: 22%, G: 56%, P: 47%), the only species isolated from all DGP wards and its ICUs were *C. tropicalis* (2%, 3%, 12%) and *Clavispora *(*Cl.*)* lusitaniae* (1%, 1%, 2%).

Despite the uneven distribution and low testing rates of some clinical isolates throughout the study periods, the isolation rate demonstrated a slight, statistically not significant increase of the NCY species (Figure 1). Nevertheless, in the DGP wards, the increase in NCA strains followed those found in the other medical specialities [15, 16, 20, 21, 26, 29, 34, 54, 55, 61, 64, 66]. A summary of the distribution of *Candida* species in epidemiological surveys of the last decades was given in [66–68]. The most frequent isolates from all the DGP ICUs (*N* = 145) were *C. albicans* (54%), *C. glabrata* (18%), *C. tropicalis* (12%), *I. orientalis* (8%), *C. parapsilosis* (3%), *Cl. lusitaniae* (3%), *Saccharomyces *(*S.*)* cerevisiae* (1%), *C. sake*, *Kluyveromyces *(*K.*)* marxianus*, and *M. guilliermondii* (0.7% each). Somewhat different isolation rates were reported for ICUs strains in France [68] and Turkey [69], where ICU isolates (France/Turkey) accounted for 57%/14% *C. albicans*, 17%/4% *C. glabrata*, 8%/28% *C. parapsilosis*, 5%/4% *I. orientalis*, and 5%/14% *C. tropicalis* and in Turkey only 3% *K. marxianus*, 2% *Wickerhamomyces anomalus*, 1% *M. guilliermondii*, 1% *C. dubliniensis*, 0.6% *Debaromyces *(*D.*)* hansenii*, and 0.3% *Clavispora lusitaniae.*

It has been reported that *C. parapsilosis* was most frequently recovered from younger patients, decreasing with age, while *C. glabrata* occurrence increased with age [70]. Whereas the frequency of *C. parapsilosis* in the DGP wards and DGP ICUs (except dermatology ICUs) were found at 16% (D), 6% (P), 2% (G), 8% G-ICU, and 3% P-ICU, *C. tropicalis* was found at 12% (P), 3% (G), 2% (D), 20% (D-ICU), 12% P-ICU, and at 8% in the G-ICU (Table 2).

Aside from *C. albicans*, the highest prevalence was found for *C. glabrata* with 17% (G), 14% (D), 9% (P), 38% (G-ICU), and 17% (P-ICU). In a Hospital-Infection-Surveillance-Study in Germany [71] evaluating nosocomial infections in the ICU, *C. albicans* was found to be the most frequently pathogen causing vascular catheter associated sepsis (5.6% in all ICUs, 2.8% in paediatric ICUs), and the fourth most agent of urinary catheter associated UTI infections (8.7%). The distribution of the yeast species in the Turkish paediatric ICU was 2%/4%/1% for *C. albicans*, *C. parapsilosis*, and *D. hansenii*. No other species from this ICU had been reported. Although the incidence of candidemia was stable over a ten-year period (0.5 episodes/10.000 patient days per year), it was five times higher in ICUs than in other surgical wards in Switzerland [72]. However, during the recent decades a progressive shift from a predominance of *C. albicans* towards NCAC/NCY species (including *C. glabrata* and *I. orientalis*) has been reported [73], with *C. glabrata* accounting for 15%–20% of infections in most countries [74–76]. These differences in the occurrences of the most important and regularly isolated yeast species in the DGP wards and ICUs of the study are demonstrated in Table 3. *C. parapsilosis* and *M. guilliermondii* are most prominent only in the dermatology wards (48%/79%) and found to a significant lesser extent in the paediatrics units (28%/18%) but not at all in the gynaecology wards. Compared to the dermatology units the levels of *C. albicans* and *C. glabrata* are about 30% higher in gynaecology and paediatrics, whereas *C. parapsilosis* occurrence is about 30% higher in dermatology and paediatrics than in gynaecology wards. The occurrence of *C. tropicalis* (Table 3) is highest in paediatric wards (68%) and significantly lower in gynaecology (14%) and dermatology (5%).

Romeo and Criseo [77] found that 8 out of their 11 *C. dubliniensis* isolates were derived from oral specimens, and only 2 were found in vaginal and one in gastric fluid. Of the 15 *C. dubliniensis* strains in this study, one was isolated from blood, 2 from sterile body fluids, 5 from dermatological, and 7 from paediatric swabs. No *C. dubliniensis*, *C. inconspicua*, and *D. hansenii* isolates were derived from DGP ICUs (Table 2) and none of these strains were found in DGP-specimens, except DGP swabs (Table 2). Gumral et al. [78] reported the lack of *C. dubliniensis* and *C. africana* strains in Turkey with vaginal *C. albicans* isolates, whilst Nnadi et al. [79] found in Nigeria no *C. dubliniensis* in vulvovaginal samples. As three *C. africana* isolates appeared in Berlin and two in Munich they were tested during a MCS in 2000, together with the strains from Angola and Madagascar. Although so far considered only as a new subspecies of *C. albicans*, *C. africana* should be reconsidered as separate species according to the original proposal of Tietz et al. [80]. This is supported by the results of Forche et al. [81] that rDNS sequences of *C. dubliniensis* differ significantly from those of *C. albicans* and that *C. africana* isolates are phylogenetically different. Moreover, *C. africana* could clearly be separated by FT-IR [82], probably nowadays by Matrix Assisted Laser-Desorption/Ionisation Time-Of-Flight Mass Spectroscopy (MALDITOF MS) as for *C. dubliniensis* [83], by pyrosequencing [84], or as described with a specific molecular method [85]. All these methods demonstrated that they are able to discriminate distinctly between the very closely related species *C. albicans*, *C. africana*, and *C. dubliniensis.* Apart from the reported isolates in the study and those from vaginal specimens from Africa [80, 81], none appeared in further German/Austrian MCS, and only a few strains were isolated later in Italy [77, 85], Spain [86], Nigeria [79], and Great Britain [84].

Protothecosis is a sporotrichosis-like infection in humans, in both immunocompromised and immunocompetent patients, and in animals. It is caused by achlorophyllic algae of the genus *Prototheca*, which belongs to the family *Chloracellae* and is rarely involved in human infections [87, 88]. The genus *Prototheca* (*P*.) consists currently of 6 species: *P. wickerhamii, P. zopfii*, *P. stagnora*, *P. ulmea*, *P. blaschkeae*, and *P. cutis*. *P. zopfii* contains currently two genotypes [89]. Species of the genus *Prototheca* exist in the environment as ubiquitous detritus inhabitants and contaminants of various substrates. General protothecosis is caused in humans mainly by *P. wickerhamii* and in domestic animals by *P. zopfii*. General symptoms are dermatitis or bovine mastitis, whereas mortal cases are extremely rare. *P. wickerhamii* and *P. zopfii* were isolated in 1998 in an outbreak in a children's unit during one of the MCS, where these organisms were transmitted from pet animals to the patients [82].

Of the twelve different species of *Malassezia* (M.) yeast described [90–92], *M. furfur* (14/0.2%), *M. globosa* (2/0.02%), *M. obtusa* (1/0.01%), *M. pachydermatis *(1/0.01%), *M. sloofiae *(1/0.01%), and *M. sympodialis *(55/3% of total, 72.3% of *M*. spp.) have been isolated during the investigations in 1998 and 1999. This parallels the report of Petry et al. [91], where *M. sympodialis* (72%) was the most frequently isolated *Malassezia* species. In addition, Petry et al. [91] reported that the back and the chest of the patients are the most common sites of the lesions, and no statistically significant difference was found between species as a function of gender, age, or the duration of the lesions [92]. *Malassezia* is strongly associated with dandruff, a common scalp disorder, although not all individuals with *Malassezia* on their skin develop dandruff. Besides *Malassezia* spp., which contribute by 5% to the population from dandruff-afflicted scalps, *Filobasidium filoforme* was reported to be the most isolated basidiomycete, whereas in healthy scalps *Cryptococcus* spp. (90%), together with *Rhodotorula mucilaginosa,* are detectable [92]. Whilst during the MCS of 1998 to 2008 *Cryptococcus neoformans* had been isolated 7 times (0.4% of total isolates), no *Rhodotorula* spp. infections were observed during the MCS. In addition, due to the limited time frame of the MCS within a study year, none of the *Exophiala*, *Malassezia*, and *Prototheca* species have been isolated anymore during the German/Austrian MCS up to 2009, with the exception of *E. dermatitidis* of which two strains each have been isolated in the MCS of 1999 and 2000 and of the two separate outbreaks of *Malassezia* and *Prototheca* spp. in 1998/99 (Table 1).

The opportunistic yeast pathogen *Trichosporon (T.) asahii, *which is part of the cutaneous fungal microbiota in humans, was isolated occasionally from 1998 to 2008 (0.2% of total isolates). *T. asahii* may be one of the routes through which deep-seated trichosporonosis is acquired, whereas environmental *T. asahii* is not associated with this infection [93].

The DGP-species distribution of urine samples was somewhat different to those isolated in a survey from 2003 to 2004 from urine specimens of 100 hospitalized patients in a Turkish hospital who had nosocomial candiduria [94].

With 80% to 95%, *C. albicans *is the predominant vaginal colonizing species in premenopausal and pregnant asymptotic and healthy women with acute *Candida* vaginitis and chronically recurrent vulvovaginal candidosis. NCAC-species, especially *C. glabrata*, are more frequent in postmenopausal, in diabetic and immunosuppressed women, paralleled by regional differences in the distribution of *Candida* species [95]. The results of this study illustrate that the yeast spectrum in gynaecological wards and its ICUs did not change significantly. This is in accordance with the findings by Mendling and Brasch [95], who reported at least for Germany no evidence of an increase of NCAC/NCY species in either acute or recurrent vaginal candidosis.

### 3.2. Specimen Distribution

The distribution of specimens according to its origin is given in Table 2. According to the different clinic specialities, the specimen types and their amount differed from those of their corresponding ICUs (Table 2). However, the ranking and type of the isolated pathogenic yeast species resembled the first five yeast pathogens from invasive fungal infections. Only *C. albicans* was found in all specimen types listed (Table 2), and *C. glabrata*, *C. parapsilosis*, and *Issatchenkia orientalis* have been isolated from most of the types of specimens at various percentages (*C. albicans*: 13%–71%; *C. glabrata*: 5%–38%; *C. parapsilosis*: 2%–19%; *C. tropicalis*: 2%–20%; *I. orientalis*: 2%–7%). All other species were differently attributed to the various specimens. The most frequent isolates from blood cultures (Table 2) were *C. albicans* (48%), *C. glabrata* (21%) *C. parapsilosis* and *C. tropicalis* (9%, each), *I. orientalis *(7%), and *C. dubliniensis* (1%), with the percentages related to the total of each individual isolate of 8%, 11%, 11%, 10%, 9%, 12%, and 7%, respectively.

As shown in Table 2, only 4 devices (0.2%) have been sent for determination of associated fungi. Three devices were derived from the dermatology and one device from the paediatric ward. From the isolated species thereof (*C. albicans*, *C. glabrata*, and *Magnusiomyces capitatus*), at least two of them belong to the numerous *Candida* species (e.g., *C. glabrata*, *C. parapsilosis*, *C. tropicalis*, *C. dubliniensis,* and *I. orientalis*), which are reported to form biofilms, catheter-related blood-stream, and device-related infections [96–98]. As it was not mandatory for the *in vitro* multicenter studies to report detailed epidemiological data, the voluntarily gathered data were insufficient to evaluate more patient-related factors.

## 4. Conclusions

About 20–25% of the world's population is affected by skin mycoses, thus being one of the most frequent forms of infection [99]. The epidemiological trend in skin mycoses worldwide is paralleled to changes of nosocomial and invasive fungal infections. Although a significant shift in the distribution of the infection causing agents is reported for dermatology, gynaecology, and paediatric wards, apart from some local breakouts with *Malassezia*, *Prototheca*, and *Exophiala* species, all the infection-causing agents have been present before and throughout the 10-year study period. Aside from significant differences in the species profiles of the DGP wards, a trend in the distribution of the DGP-species could not be detected and the overall aetiology has not changed during the time period of the multicenter studies from 1997 to 2009. But, besides the typical skin pathogens like *C. albicans*, *C. glabrata*, *C. parapsilosis*, *C. tropicalis*, *C. dubliniensis*, and *C. inconspicua,* infections with atypical, rare, or “cryptic” yeast isolates, which all have been existent, such as *Issatchenkia orientalis*, *Saccharomyces cerevisiae*, *Meyerozyma guilliermondii*, *Kluyveromyces marxianus*, *Clavispora lusitaniae*, *Debaromyces hansenii*, and *Yarrowia lipolytica,* tend also to emerge in the DGP wards and DGP ICUs, respectively, as reported for the other wards. This may be markedly amplified by the taxonomic changes which are to be implemented since the beginning of 2013 comprising taxonomic reclassifications and concomitant (partially) renaming of various species according to the “one fungus one name” principle. However, this novel practice based on the phylogenetic mapping of the species may allow in future a better association of different or similar pathogens to clinical entities and characteristics. This may also lead to a better and reliable assessment of *in vitro* susceptibility data (given in a corresponding paper [46]), which represent the basis not only for specific antifungal therapy, but in particular also for calculated (“empiric”) antifungal therapy.

## Figures and Tables

**Table 1:** Yeast species distribution listed according to their isolation-frequency during the multicenter studies from 1998 to 2008 (% of individual species), together with their reported and current valid genus and species denominations (empty spaces = no isolates recovered), in comparison to a general German yeast species-distribution survey (2008^b^) and a survey on yeast distribution in a dermatologic clinic (2002–2011^c^).

Genus and species denominations		Year isolated/tested																		Surveys from Germany			
Yeast name reported	Current valid name^a^	1998–2008		1998		1999		2000		2001		2002		2003		2004		2008		2008^b^		2003–2011^c^	
		*N* = 1,862		N = 222		N = 117		N = 259		N = 277		N = 535		N = 172		N = 76		N = 204		*N* = 15,616		*N* = 3,402	
		*n*	%	*n*	%	*n*	%	*n*	%	*n*	%	*n*	%	*n*	%	*n*	%	*n*	%	*n*	%	*n*	%
*Candida albicans *	*Candia albicans *	840	45.1	57	25.7	2	1.7	222	85.6	45	16.3	251	46.9	104	60.5	41	54.0	118	57.8	8,718	55.8	2,223	65.3
*Candida glabrata *	*Candida glabrata *	266	14.3	18	8.1					111	40.7	77	14.4	23	13.4	5	6.6	32	15.7	2,171	13.9	169	5.0
*Candida tropicalis *	*Candida tropicalis *	133	7.1	9	4.1					41	14.8	63	11.8	7	4.1	5	6.6	8	3.9	532	3.4	20	0.6
*Candida parapsilosis *	*Candida parapsilosis *	121	6.5	52	23.4					16	5.8	28	5.2	1	0.6	6	7.9	18	8.8	292	1.9	395	11.6
*Candida krusei *	*Issatchenkia orientalis *	118	6.3	8	3.6					26	9.4	47	8.8	25	14.5	4	5.3	8	3.9	370	2.4	27	0.8
*Saccharomyces cerevisiae *	*Saccharomyces cerevisiae *	41	2.2	1	0.5	4	3.4			11	4.0	16	3.0	6	3.5	2	2.6	1	0.5	154	1.6	10	0.3
*Candida guilliermondii *	*Meyerozyma guilliermondii *	33	1.8	22	9.9					2	0.7	5	0.9			1	1.3	3	1.5	25	0.2	123	3.6
*Candida africana *	*Candida africana *	32	1.7					32	12.4														
*Candida lusitaniae *	*Clavispora lusitaniae *	28	1.5	6	2.7					5	1.8	10	1.9			1	1.3	6	2.9	103	0.7	9	0.3
*Candida kefyr *	*Kluyveromyces marxianus *	25	1.3	1	0.5	1	0.9			3	1.1	13	2.4	2	1.2	5	6.6			83	0.9	31	0.9
*Candida dubliniensis *	*Candida dubliniensis *	15	0.8					3	1.2	2	0.7	2	0.4			2	2.6	6	2.9	215	1.4		
*Candia sake *	*Candia sake *	9	0.5							5	1.8	4	0.8							10	0.06	4	0.1
*Geotrichum capitatum *	*Magnusiomyces capitatus *	6	0.3							2	0.7	4	0.8							13	0.01		
*Candida inconspicua *	*Candida inconspicua *	5	0.3	1	0.5							3	0.6			1	1.3			6	0.04	4	0.2
*Candida norvegensis *	*Pichia norvegensis *	5	0.3							1	0.4	4	0.8							10	0.06	3	0.1
*Candida famata *	*Debaromyces hansenii *	4	0.2									2	0.4					2	1.0	16	0.2	33	1.0
*Candida lipolytica *	*Yarrowia lipolytica *	4	0.2	1	0.5					1	0.4	1	0.2					1	0.5	8	0.1	9	0.3
*Candida maritima *	*Candida maritima *	2	0.1											2	1.2								
*Galactomyces geotrichum *	*Geotrichum candidum *	2	0.1							1	0.4	1	0.2							47	0.5	8	0.2
*Candida ciferrii *	*Trichomonascus ciferrii *	2	0.1							1	0.4	1	0.2										
*Candida membranifaciens *	*Candida membranifaciens *	1	0.07							1	0.4									1	0.01		
*Pichia cactophila *	*Pichia cactophila *	1	0.1															1	0.5	0	0		
*Candida fermentans *	*Pichia fermentans *	1	0.1									1	0.1							1	0.01		
*Exophiala dermatitidis *	*Exophiala dermatitidis *	51	2.7	36	16.2	11	9.4	2	0.8	2	0.7									26	0.3		
*Malassezia *spp.	*Malassezia *spp.	76	4.1	7	3.2	69	58.9															115	3.4
*Cryptococcus neoformans *	*Cryptococcus neoformans *	7	0.4	2	0.9							2	0.4			3	4.0			1	0.01	3	0.1
*Trichosporon asahii *	*Trichosporon asahii *	3	0.2							1	0.4			2	1.2					5	0.03	26	0.8
*Trichosporon mucoides *	*Trichosporon mucoides *	1	01	1	0.5															2	0.01	101	3.0
*Prototheca zopfii *	*Prothoteca zopfii *	25	1.3			25	21.4																
*Prototheca wickerhamii *	*Prothoteca wickerhamii *	5	0.3			5	4.3																

^a^According to SpeciesFungorum [47] and Mycobank [48].

^b^Survey of 18 centres, consecutive clinical yeast isolates (all wards) in one quarter of 2008, in parallel to a MCS in 2008 [49].

^c^Only yeast isolates from the Dermatology Department of the Charité, Berlin.

**Table 2:** Isolation rates (number/%) of DGP-associated yeast species in relation to their origin (clinical speciality and specimen), which have been recovered during the multicenter studies from 1998 to 2008.

Isolate origin	Frequency (total = 1,862)	Dermatology, gynaecology, and paediatrics-associated clinical yeast species, isolated during the multicentre studies from 1998 to 2008																			
		*Candida albicans *	*Candida dubliniensis *	*Candida glabrata *	*Candida inconspicua *	*Candida parapsilosis *	*Candida tropicalis *	Other* Candida *spp*. *	*Clavispora lusitaniae *	*Debaromyces hansenii *	*Geotrichum candidum *	*Issatchenkia orientalis *	*Kluyveromyces marxianus *	*Magnusiomyces capitatus *	*Meyerozyma guilliermondii *	*Pichia* spp.	*Saccharomyces cerevisiae *	*Trichomonascus ciferrii *	*Yarrowia lipolytica *	*Cryptococcus neoformans *	*Trichosporon *spp.
	*N* (%)	840	15	266	5	121	133	43*	28	4	2	118	25	6	33	7	41	2	4	7	4
		(**45.1**)	(**0.8**)	(**14.3**)	(**0.3**)	(**6.5**)	(**7.1**)	(**2.3**)	(**1.5**)	(**0.2**)	(**0.1**)	(**6.3**)	(**1.3**)	(**0.3**)	(**1.8**)	(**0.4**)	(**2.2**)	(**0.1**)	(**0.2**)	(**0.4**)	(**0.2**)
Paediatrics	767 (**41.2**)	358 (**47**)	10 (**1**)	108 (**14**)	1 (**0.1**)	46 (**6**)	91 (**12**)	39 (**5**)	15 (**2**)	1 (**0.1**)	0	58 (**8**)	13 (**2**)	4 (**0.5**)	6 (**0.8**)	5 (**0.7**)	3 (**0.4**)	0	1 (**0.1**)	4 (**0.5**)	4 (**0.5**)
Gynaecology	578 (**31.0**)	321 (**56**)	0	99 (**17**)	4 (**0.7**)	12 (**2**)	18 (**3**)	32 (**6**)*	7 (1)	1 (**0.2**)	2 (**0.4**)	37 (**6**)	8 (**1**)	0	0	0	35 (**6**)	0	2 (**0.4**)	0	0
Dermatology	372 (**20.0**)	83 (**22**)	5 (**1**)	33 (**9**)	0	58 (**16**)	7 (**2**)		3 (**0.8**)	2 (**0.5**)	0	12 (**3**)	3 (**0.8**)	2 (**0.5**)	26 (**7**)	2 (**0.5**)	1 (**0.3**)	2 (**0.5**)	1 (**0.3**)	3 (**1**)	0
ICU Paediatrics	127 (**6.8**)	70 (**55**)	**0**	21 (**17**)	0	4 (**3**)	15 (**12**)	1 (**0.8**)	1 (**0.8**)	0	0	11 (**9**)	1 (**0.8**)	0	1 (**0.8**)	0	2 (**2**)	0	0	0	0
ICU Gynaecology	13 (**0.7**)	5 (**38**)	0	5 (**38**)	0	1 (**8**)	1 (**8**)	0	1 (**8**)	0	0	0	0	0	0	0	0	0	0	0	0
ICU Dermatology	5 (**0.3**)	3 (**60**)	0	0	0	0	1 (**20**)	0	1 (**20**)	0	0	0	0	0	0	0	0	0	0	0	0
Sterile fluid	204 (**11.0**)	109 (**53**)	2 (**1**)	28 (**14**)	0	13 (**6**)	24 (**12**)	1 (**0.5**)	7 (**3**)	0	0	9 (**4**)	3 (**2**)	1 (**0.5**)	0	0	2 (**1**)	0	0	5 (**3**)	0
Blood culture	138 (**7**)	66 (**48**)	1 (**0.7**)	29 (**21**)	0	13 (**9**)	13 (**9**)	0	1 (**0.7**)	1 (**0.7**)	0	10 (**7**)	0	0	0	0	0	0	0	0	0
Catheter	31 (**1.7**)	22 (**71**)	0	2 (**7**)	0	5 (**16**)	1 (**3**)	0	0	0	0	1 (**3**)	0	0	0	0	0	0	0	0	0
Aspirate	19 (**1.0**)	9 (**47**)	0	1 (**5**)	0	0	3 (**16**)	3 (**16**)	0	0	0	0	2 (**11**)	0	0	0	1 (**1**)	0	0	5 (**1**)	0
Device	4 (**0.2**)	2 (**50**)	0	1 (**25**)	0	0	0	0	0	0	0	0	0	1 (**25**)	0	0	0	0	0	0	0
Material, dermatologic	228 (**12.2**)	30 (**13**)	0	0	0	35 (**16**)	0	0	0	2 (**1**)	0	0	0	0	15 (**11**)	0	0	0	0	0	0
Material, gynaecologic	146 (**7.8**)	88 (**61**)	0	37 (**26**)	1 (**1**)	5 (**4**)	6 (**4**)	0	0	0	0	3 (**2**)	0	0	0	0	4 (**3**)	0	1 (**1**)	0	0
Material, solid	111 (**6.0**)	45 (**40**)	0	9 (**8**)	0	9 (**8**)	6 (**6**)	1 (**1**)	0	0	0	3 (**3**)	0	0	3 (**3**)	1 (**1**)	3 (**3**)	0	0	1 (**1**)	0
Urine	21 (**1.1**)	9 (**43**)	0	3 (**14**)	0	1 (**5**)	0	0	1 (**5**)	0	0	3 (**14**)	4 (**19**)	0	0	0	0	0	0	0	0
Stool	20 (**1.1**)	13 (**65**)	0	6 (**30**)	0	0	0	0	0	0	0	1 (**5**)	0	0	0	0	0	0	0	0	0
Swabs, paediatric	460 (**24.7**)	214 (**47**)	7 (**2**)	66 (**14**)	0	11 (**2**)	68 (**15**)	5 (**1**)	7 (**2**)	0	0	55 (**12**)	6 (**1**)	3 (1)	4 (**1**)	5 (**1**)	3 (**1**)	0	1 (**0.2**)	0	4 (**1**)
Swabs, gynaecologic	364 (**19.6**)	189 (**52**)	0	55 (**15**)	3 (**1**)	7 (**2**)	6 (**2**)	1 (**0.3**)	8 (**2**)	0	2 (**1**)	26 (**7**)	7 (**2**)	0	0	0	27 (**7**)	0	1 (**0.3**)	0	0
Swabs, dermatologic	116 (**6.2**)	44 (**38**)	5 (**4**)	21 (**18**)	0	22 (**19**)	6 (**5**)	2 (**2**)	4 (**3**)	0	0	7 (**6**)	0	2 (**2**)	1 (**1**)	1 (**1**)	1 (**1**)	0	1 (**1**)	0	0

Other *Candida* spp.: *C. maritima, C. membranifaciens, C. sake, *and* Candida africana; Pichia*spp.*: P. cactophila, P. fermentans, *and* P. norvegensis; Trichosporon *spp.*: T. asahii* and* T. mucoides*.

Aspirate: bursa, limbic, pericardial, transtracheal, pus, and pleura.

Catheter: indwelling, urine, vascular, venereal, ports, and anaesthetic tube.

Sterile fluid: sterile body fluids, liquor, bile, dialysates, drainage, BAL, tracheal secrets, pleura, lachrymal, synovial, and serum, except blood and urine.

Solid (sterile) material: tissues/lung tissue, bone-marrow, throat discharge/sputum, abscess, spleen, bone, liver, stomach, and ear.

Device: contact lenses, heart valves, stents, pacemakers, and artificial joints.

Dermatological material: skin, nails, hairs, dandruff, and scales.

Gynaecological material: scrapings, genital-, prostate secrets, and ejaculate.

Urine: mid-stream, punctuate, and catheter.

**Table 3:** Occurrence differences, respectively, of profiles of the most frequent isolated yeast species (number/percentage (% of species)) in the DGP wards and DGP ICUs.

Ward/ICU	*N* (total)	*C. albicans * (**840**)	*C. glabrata*(**266**)	*C. parapsilosis*(**121**)	*C. tropicalis* (**133**)	*M. guilliermondii*(**33**)
Dermatology	372	83/22.3 (**9.9**)	33/8.9 (**12.4)**	58/15.6 (**47.9**)	7/1.9 (**5.3**)	26/7.0 (**78.8**)
Gynaecology	578	321/55.5 (**38.2**)	99/17.1 (**37.2**)	12/2.1 (**9.9**)	18/3.1 (**13.5**)	**0**
Paediatrics	767	358/46.7 (**42.6**)	108/14.1 (**40.6**)	46/6.0 (**38.0**)	91/11.9 (**68.4**)	6/0.8 (**18.2**)
D-ICU	5	3/60.0 (**0.4**)	**0**	**0**	1/20.0 (**0.8**)	**0**
G-ICU	13	5/38.5 (**0.6**)	5/38.5 (**1.9**)	1/7.7 (**0.8**)	1/7.7 (**0.8**)	**0**
P-ICU	127	70/55.1 (**8.3**)	21/16.5 (**7.9**)	4/3.2 (**3.3**)	15/11.8 (**11.3**)	1/0.8 (**3.0**)
